# Effects of Initial pH and Carbonate Rock Dosage on Bio-Oxidation and Secondary Iron Mineral Synthesis

**DOI:** 10.3390/toxics11030224

**Published:** 2023-02-27

**Authors:** Yuran Fu, Ruixue Zhang, Neng Wang, Pan Wu, Yahui Zhang, Li An, Yuhao Zhang

**Affiliations:** 1 Resource and Environmental Engineering College, Guizhou University, Guiyang 550025, China; 2 Key Laboratory of Karst Georesources and Environment, Ministry of Education, Guiyang 550025, China

**Keywords:** pH, *A. ferrooxidans*, acid mine drainage, carbonate rocks, secondary iron minerals

## Abstract

The effect of pH is a key factor in biomineralization mediated by *Acidithiobacillus ferrooxidans* to promote the transformation of Fe into secondary iron minerals. This study aimed to investigate the effects of initial pH and carbonate rock dosage on bio-oxidation and secondary iron mineral synthesis. Variations in pH and the concentrations of Ca^2+^, Fe^2+^, and total Fe (TFe) in the growth medium of *A. ferrooxidans* were examined in the laboratory to determine how they affect the bio-oxidation process and secondary iron mineral synthesis. The results showed that in systems with an initial pH of 1.8, 2.3, and 2.8, the optimum dosages of carbonate rock were 30, 10, and 10 g, respectively, which significantly improved the removal rate of TFe and the amount of sediments. At an initial pH of 1.8 and a carbonate rock dosage of 30 g, the final removal rate of TFe reached 67.37%, which was 28.03% higher than that of the system without the addition of carbonate rock, and 36.9 g·L^−1^ of sediments were generated, which was higher than that of the system without the addition of carbonate rock (6.6 g·L^−1^). Meanwhile, the number of sediments generated by adding carbonate rock were significantly higher than those without the addition of carbonate rock. The secondary minerals were characterized by a progressive transition from low crystalline assemblages composed of calcium sulfate and subordinated jarosite, to well crystal-line assemblages composed of jarosite, calcium sulfate, and goethite. These results have important implications for comprehensively understanding the dosage of carbonate rock in mineral formation under different pH conditions. The findings help reveal the growth of secondary minerals during the treatment of AMD using carbonate rocks under low-pH conditions, which offers valuable information for combining the carbonate rocks with secondary minerals to treat AMD.

## 1. Introduction

*Acidithiobacillus ferrooxidans* is widely distributed in acid mine drainage (AMD). This Gram-negative bacterium can grow in a wide range of temperatures (4–37 °C) and acidic conditions (pH 1.0–4.5), but its optimal growth pH is about 2.0 [1]. Numerous studies have shown that the oxidation rate of Fe^2+^ can be increased by 10^5^ to 10^6^ times by *A. ferrooxidans* [2,3], and secondary minerals formed by hydrolysis of Fe^3+^, such as schwertmannite, jarosite, and goethite, with their relatively high availability, specific crystal structures, and large surface areas facilitates their use as adsorbents for remediating wastewater contaminated with heavy metals [4,5]. Acidophilic organisms contribute to the precipitation of secondary iron minerals in the modern sediments of the river [6,7]. It confirms that the presence of biological nucleation sites (cell walls of bacteria or fungi) can modify the expected mineral precipitation schemes offered by the bulk physicochemical conditions in which microorganisms grow. The extracellular polymeric substances (EPS) released by the microorganisms can serve as nucleation sites for biosynthesis of the secondary iron minerals process [8,9]. *A. ferrooxidans* plays an important role in the biosynthesis of secondary iron minerals, and particularly in improving the self-purification ability of the environment, thus enhancing environmental and economic benefits in AMD treatment [10]. 

The pH of a solution has a major influence on microbial activities and the oxidation rates of Fe^2+^ [11,12]. Unsuitable pH environments are harmful to the growth and metabolism of bacteria, resulting in a decline in bacterial oxidation [13]. *A. ferrooxidans* can substantially alter the pH conditions of the bacteria−mineral interface and its surrounding microenvironment, consequently enhancing the mineral dissolution process [14]. Under different pH and Eh conditions, microorganisms can induce the formation of secondary minerals with different morphologies, structures, and particle sizes [15,16]. The formation of secondary iron minerals depends on the content of hydroxyl complexes or the transformation of the monomers to polymers, which are greatly affected by the pH of a solution [17]. It is generally accepted that natural schwertmannite formation commonly occurs in Fe-rich sulfate solutions in the pH range of 2.50–4.50 with a lower or higher pH promoting either jarosite or ferrihydrite/goethite precipitation, respectively [18]. These secondary minerals will naturally passivate the heavy metals in water by absorption or co-precipitation, which effectively remove heavy metals from AMD [19]. It seemed that pH was a crucial factor in determining the formation of secondary minerals.

Globally, karst-dominated landscapes cover 10–15% of the land area and are typically characterized by rich carbonate rocks [20]. An increase in coal mining activities in karst areas produces considerable amounts of AMD, which is characterized by low pH, high concentrations of iron (mostly Fe^2+^), and other trace elements [21]. Considering the severe adverse effects of AMD on the fragile ecological environment of karst areas [22,23], the control and management of AMD have practical significance for the protection of ecological environments and sustainable development in karst watersheds. The AMD passive treatment technology was developed with cheap and easily available carbonate rocks as the reactive medium to effectively improve the pH of AMD, and it has had a good removal effect on Fe [24]. Additionally, the production of secondary minerals plays an important role in the removal of other heavy metals from AMD [25]. Our previous research has shown that the combined treatment of AMD, with biological oxidation and carbonate rocks under low-pH conditions, is a potential treatment technique [26]. The feasibility to pursue sequential and fractional secondary minerals is a driving factor that promotes this approach. However, it is still necessary to determine how the reaction processes differ based on pH, thereby impacting the combined mechanism of action of carbonate rocks and biological oxidation.

Therefore, the objectives of this study are to: (1) investigate effects of carbonate rock dosage and initial pH on the rates of pH rise; (2) explore the effect of pH regulation on the reaction processes of carbonate rock and biological oxidation in acidic environments; (3) select an optimal dosage of carbonate rocks based on initial pH to promote the synthesis of secondary iron minerals. This study will be beneficial for the rational selection of carbonate rocks, and it can be used to extend the application of low-cost and regenerable secondary iron minerals for AMD treatment in karst areas.

## 2. Materials and Methods

### 2.1. Preparation of A. ferrooxidans and Carbonate Rock Samples

The leachate from a coal mine was inoculated in a modified 9K liquid medium which consisted of FeSO_4_·7H_2_O (99.9%, Sinopharm Group Co., Ltd., Shanghai, China) 44.24 g·L^−1^, (NH_4_)_2_SO_4_ (≥99.0%, Sinopharm Group Co., Ltd., Shanghai, China) 3 g·L^−1^, KCl (≥99.0%, Sinopharm Group Co., Ltd., Shanghai, China) 0.1 g·L^−1^, K_2_HPO_4_ (≥98.0%, Tianjin Kemiou Chemical Reagent Co., Ltd., Tianjin, China) 0.5 g·L^−1^, Ca(NO_3_)_2_·4H_2_O (≥99.0%, Tianjin Kemiou Chemical Reagent Co., Ltd., Tianjin, China) 0.01 g·L^−1^, and MgSO_4_·7H_2_O (≥98.0%, Tianjin Kemiou Chemical Reagent Co., Ltd., Tianjin, China) 0.5 g·L^−1^ at 20% [27], and the mixture was then placed in a digital-display air thermostat oscillator (ZD-85A, China) at 30 °C and 160 r·min^−1^ for 3–4 d until the color of the mixture in the reaction system changed from green to red–brown. After multiple enrichments and cultures, an *A. ferrooxidans* bacterial solution was obtained. The purity of all reagents was analytical grade. The purified *A. ferrooxidans* bacterial solution was identified by metagenomic microbial classification, sequenced, and preserved at 4 °C.

**Figure 1:**
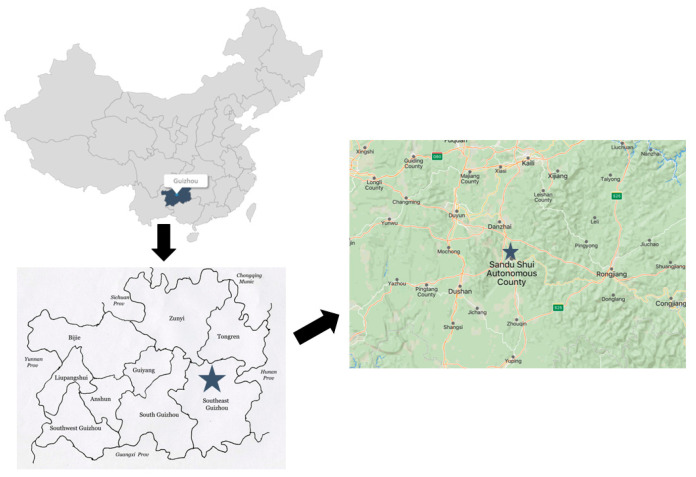
Sketch map of Sandu County, Buyi and Miao Autonomous Prefecture of Qiannan, Guizhou Province.

The carbonate rocks used in this experiment were mainly comprised of calcite and dolomite (95.7%), and collected from Sandu County, Buyi and Miao Autonomous Prefecture of Qiannan, Guizhou Province (Figure 1) [28]. They were ground and screened to different particle sizes, washed, and dried.

### 2.2. Experimental Methods

The carbonate rocks (Φ1.3–1.6 cm) were repeatedly washed with deionized water and dried in an oven at 50 °C for 8 h. A total of 15 mL of *A. ferrooxidans* LX5 inoculum was used to inoculate Erlenmeyer flasks with different amounts of carbonate rocks and 135 mL of a modified 9 K liquid medium stock solution. Treatment systems with different initial pH levels were set up, and three parallel experiments (treatments ①, ②, and ③) were established for each system (Table 1). The meaning of the labels in each figure in this study can be found in Table 1.

### 2.3. Measurement Methods and Data Processing

The pH was determined using a pHS-3C digital pH-meter, Fe^2+^ was measured using the 1,10-phenanthroline method, and then TFe and Ca^2+^ contents were determined by atomic absorption spectrometry (TAS-990, Beijing Purkinje General Instrument Co., Ltd., Beijing, China). The mineral phases were analyzed with X-ray diffraction (XRD) using CuKα radiation (40 KV, 40 mA). The samples were scanned from 10° to 70° 2*θ* with steps of 0.02°. The characteristic reflection peaks of the minerals were compared with those in the data files of the Joint Committee on Powder Diffraction Standards (JCPDS). The surface morphologies of the carbonate rocks and sediments were directly observed using thermal field-emission scanning electron microscopy (SEM, JSM-7001F, JEOL Ltd., Japan).

Microsoft Excel (2019) was used for the statistical analysis of the data obtained in this experiment, and the XRD analysis was conducted using the Jade 6.5 software. All the experimental data were represented by the mean value and standard deviation, with related diagrams drawn using Origin 8.0.

In this study, the oxidation rate (*OR*) of Fe^2+^ and removal rate (*RR*) of TFe were calculated using Equations (1) and (2), where C(Fe^2+^) and C(TFe)_0_ denote the metal concentrations at the beginning and C(Fe^2+^)_t_ and C(TFe)_t_ denote the metal concentrations at different incubation times.

(1) $$OR=\frac{C_{{(\mathrm{Fe}}^{2+})0}{\;-\;C}_{{(\mathrm{Fe}}^{2+})t}}{C_{{(\mathrm{Fe}}^{2+})0}}\times 100\%$$

(2) $$RR=\frac{{(C}_{\left(\mathrm{TFe}\right)0}\;{-\;C}_{\left(\mathrm{TFe}\right)t})}{C_{\left(\mathrm{TFe}\right)0}}\times 100\%$$

## 3. Results and Discussion

### 3.1. Variations of pH and Ca^2+^ Concentrations in Each System

The biomineralization process involving *A. ferrooxidans* included two chemical reactions: (1) Fe^2+^ biological oxidation, where the consumption of H^+^ increases pH (Equation (3)) [29], and (2) hydrolysis of Fe^3+^ to form secondary minerals, such as schwertmannite and jarosite, where the release of H^+^ reduces pH (Equations (4) and (5)) [30]. In karst areas, AMD is neutralized by the surrounding carbonate rocks in the early stages of AMD formation (Equation (6)) [28]:

(3) $${\mathrm{Fe}}^{2+}{+1/4\;O}_{2}{+H}^{+}\to {\mathrm{Fe}}^{3+}+1/2{\;H}_{2}O\;(\text{A. ferrooxidans})$$

(4) $${8\mathrm{Fe}}^{3+}{+14H}_{2}{O+\mathrm{SO}}_{4}{}^{2-}\to {\;\mathrm{Fe}}_{8}O_{8}{\left(\mathrm{OH}\right)}_{6}{\mathrm{SO}}_{4}\left(\mathrm{Schwertmannite}\right){+22H}^{+}$$

(5) $${{(K,\mathrm{Na},\mathrm{NH}}_{4}{,H}_{3}O)}^{+}{+3\mathrm{Fe}}^{3+}{+2\mathrm{SO}}_{4}{}^{2-}{+6H}_{2}O\to \;{(K,\mathrm{Na},\mathrm{NH}}_{4}{,H}_{3}{O)\mathrm{Fe}}_{3}{{(\mathrm{SO}}_{4})}_{2}{\left(\mathrm{OH}\right)}_{6}\left(\mathrm{Jarosite}\right){+6H}^{+}$$

(6) $${\mathrm{CaCO}}_{3}{+2H}^{+}\to {\;\mathrm{Ca}}^{2+}{+\mathrm{CO}}_{2}{+H}_{2}O$$

**Figure 2:**
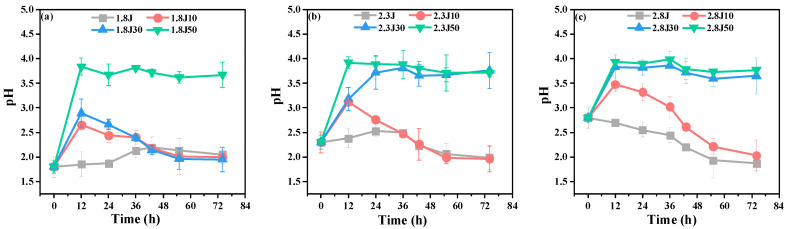
Variation in the pH values in each system from an initial pH of (**a**) 1.8; (**b**) 2.3; and (**c**) 2.8.

When carbonate rocks were added to the systems, the pH of 2.8J decreased gradually at the beginning of the experiment (Figure 2), while the pH of the other systems rose quickly from 0–12 h, and then decreased. The results indicated that Fe^3+^ hydrolyzed more easily at a higher initial pH. Based on Equations (3) and (6), the pH values of the systems with carbonate rocks increased faster than those without carbonate rocks. Some scholars found that the active radicals (for example, •OH) were produced when the pH value was increased by carbonate rocks in the process of base release, which accelerated the oxidation of Fe^2+^ and hydrolytic precipitation of Fe [31,32]. pH = 2.50–4.50 is conducive for the efficient activity of *A. ferrooxidans*, and the formation of secondary iron minerals [33]. The pH values of the systems with carbonate rocks could be maintained at 2.50–4.50. Therefore, based on the initial pH value, the number of carbonate rocks added can promote the formation and transformation of secondary minerals. Additionally, the activity of *A. ferrooxidans* was inhibited in low-pH environments, which slowed the oxidation rate of Fe^2+^. For example, the pH of 1.8J was lower than that of 2.3J. According to Equations (4) and (5), during the synthesis of schwertmannite, 1 mol Fe^3+^ hydrolysis could release 2.75 mol H^+^, while during the synthesis of jarosite, 1 mol Fe^3+^ hydrolysis could release 2 mol H^+^ [34]. Compared with the other systems, the pH values of 1.8J30, 2.3J10, and 2.8J, at the end of experiment, were less than 2 because more H^+^ was being released. 

**Figure 3:**
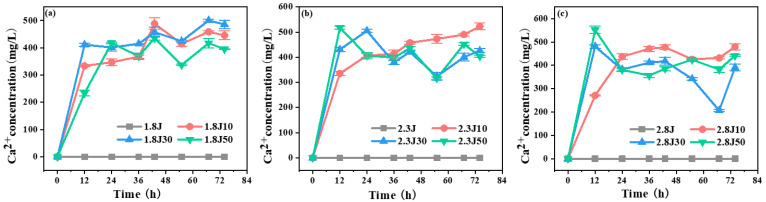
Variation in Ca^2+^ concentrations in each system from an initial pH of (**a**) 1.8; (**b**) 2.3; and (**c**) 2.8.

High concentrations of Ca^2+^ slowed the transformation of ferrihydrite into other ferric minerals because of carbonate rock dissolution, as indicated by the Ca^2+^ concentration during the experimental process (Figure 3). For all dosages tested, the dissolution of carbonate rocks was rapid in the initial 12 h and fluctuated at later stages. The fluctuation in Ca^2+^ in each system was strong because of calcium sulfate production. The pH values of 1.8J50, 2.3J30, 2.3J50, 2.8J30, and 2.8J50 increased rapidly during 0–12 h, began to decrease slightly at 36 h, and then were maintained at 3.59–3.76 during 55–74 h. Slight fluctuations in pH lead to changes in the CaCO_3_-H_2_O system [35]. Practically, Ca in the solution plays a role in increasing the pH value of the wastewater. Masindi et al. found that the Ca in struvite matrices will react with AMD water to release hydroxyl ions (OH^−^) which then contribute to an increase in pH, and higher pH could result in absorption or co-precipitation of different contaminants from AMD, thus leading to the reduction of the levels in the product water [36].

### 3.2. Oxidation Rate of Fe^2+^ and Removal Rate of TFe in Each System

**Figure 4:**
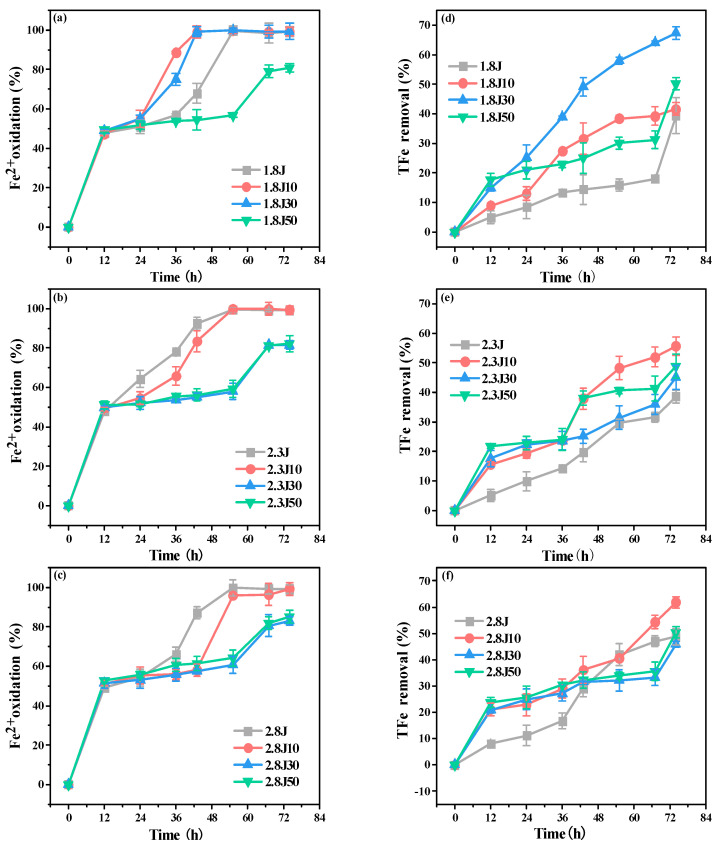
Fe^2+^ oxidation rates (**a**–**c**) and total Fe precipitation (**d**–**f**) at pH 1.8, 2.3, and 2.8, respectively, under different dosages of carbonate rock.

Excessive pollutants in AMD lead to continuous declines in water quality in karst areas. Improving the efficiency of Fe^2+^ oxidation and subsequent Fe^3+^ hydrolysis are key steps for AMD treatment. The oxidation rate of Fe^2+^ in each system (Figure 4a–c) displayed an approximately increasing linear trend from 0 to 12 h. The oxidation rates of Fe^2+^ in 1.8J10 and 1.8J30 reached 99% at 43 h, and were completely oxidized at 55 h in 1.8J, 2.3J, 2.3J10, 2.8J, and 2.8J10. The different patterns of Fe^2+^ oxidation can be primarily ascribed to variations in pH. An appropriate pH exists for the bio-oxidation of Fe^2+^ to promote the formation and phase transformation of secondary iron minerals. For example, when the initial pH was 1.8, 10 g or 30 g of carbonate rocks (2.18 < pH < 2.66) significantly accelerated the Fe^2+^ oxidation process. However, the degree of inhibition of Fe^2+^ oxidation caused by secondary iron minerals covering *A. ferrooxidans* was stronger than that of Fe^2+^ oxidation indirectly caused by the addition of carbonate rocks, which hindered the conversion of Fe^2+^ to Fe^3+^ [37]. The final oxidation rates of Fe^2+^ among the systems were relatively low with the additions of 30 g and 50 g of carbonate rocks. Zhou et al. found that the bio-oxidation of Fe^2+^ was slowed down by adding OH^−^ into the system for pH-control in the later stage [9].

The TFe removal rate in each treatment gradually increased (Figure 4d–f). The final removal rate of TFe in the 1.8J30 system was 67.37%, being 28.03% higher than that in 1.8J (39.34%), and the final removal rates of TFe in 2.3J10 and 2.8J10 were 55.63% and 61.82%, respectively, being 16.99% and 13.1% higher than those in 2.3J (38.64%) and 2.8J (48.72%), respectively. Therefore, the optimum dosages of carbonate rocks for the systems with initial pH values of 1.8, 2.3, and 2.8 were 30, 10, and 10 g, respectively. The addition of Fe^3+^ boosts the growth of *A. ferrooxidans* and eliminates the effect of low pH on its activity [38]. Thus, the addition of an appropriate amount of carbonate rocks accelerated the oxidation of Fe^2+^ and increased the content of Fe^3+^ in the solution, which was beneficial for increasing the activity of *A. ferrooxidans* and promoting the formation of secondary minerals (Equations (4) and (5)). 

### 3.3. Characterization of the Sediments and Secondary Iron Mineral Phases for Each System

The oxidation of Fe^2+^ by *A. ferrooxidans*, and the combination of dissolved metals, resulted in the formation of a large quantity of precipitates [39]. 

**Figure 5:**
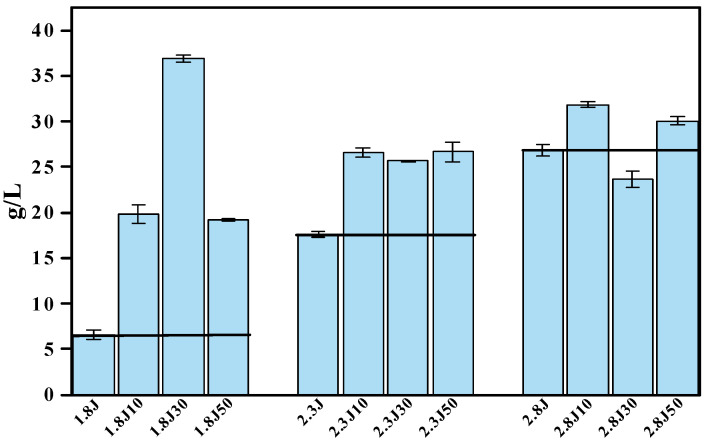
Amount of sediment produced in the different systems at pH 1.8, 2.3, and 2.8 under different dosages of carbonate rock.

The amounts of sediment produced in the different systems (Figure 5), with the addition of carbonate rocks, were significantly higher than those in the absence of carbonate rocks. The 1.8J30, 2.8J10, and 2.8J10 were the highest sediments produced in different initial pH systems, respectively. The treatment 1.8J30 generated 36.9 g·L^−1^ of sediment, which was higher than that in 1.8J (6.6 g·L^−1^). The reasons for the increase in mineral mass include: (1) the addition of carbonate rocks resulted in an optimal environment conducive to the growth of *A. ferrooxidans*, thereby promoting the synthesis of secondary iron minerals and the formation of new phases; (2) the existence of crystal seeds (including jarosite and CaSO_4_·2H_2_O) provided nucleation sites for mineral formation, thus eliminating the induction period, accelerating the initial precipitation of minerals, and increasing mineral production [40,41,42]. 

**Figure 6:**
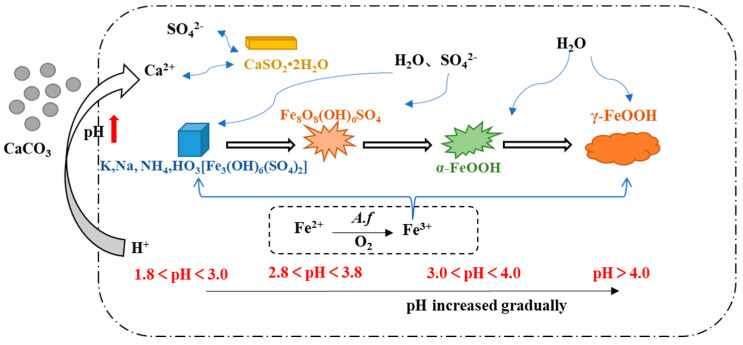
Influence of pH changes on secondary mineral formation during the interaction between AMD and carbonate rock.

**Figure 7:**
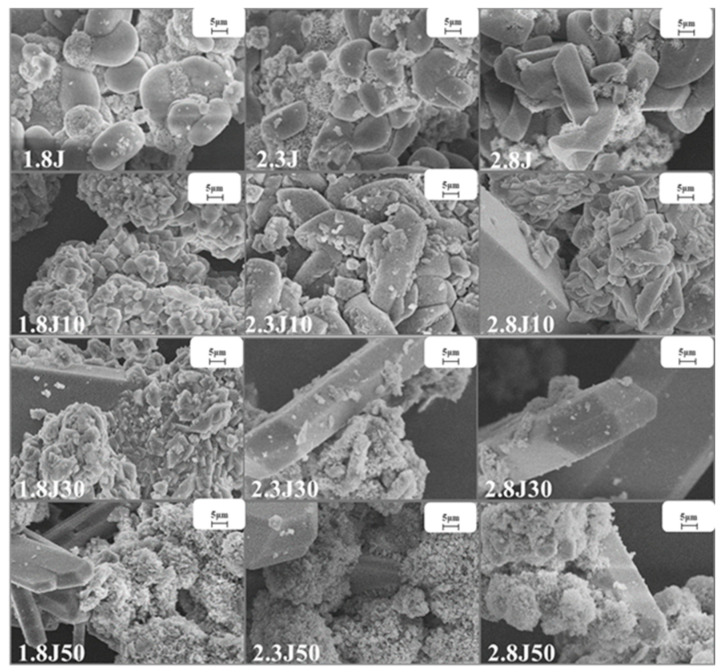
SEM images of sediments collected from the different systems at 1.8, 2.3, and 2.8 under different dosages of carbonate rock.

**Figure 8:**
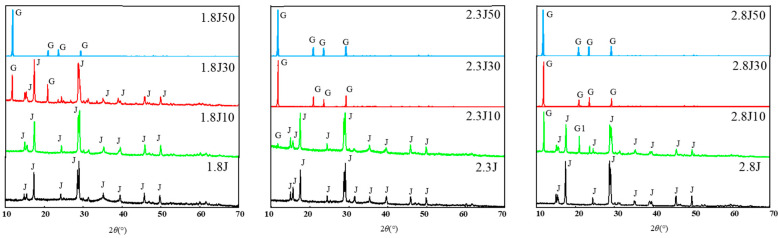
XRD patterns of sediments in the different systems at pH 1.8, 2.3, and 2.8 under different dosages of carbonate rock: J: jarosite, G: calcium sulfate, G1: goethite.

Under various pH conditions, different iron minerals, such as schwertmannite, jarosite, and goethite, were precipitated from AMD (Figure 6). The XRD patterns and SEM images of the sediments are shown in Figure 7 and Figure 8, respectively. According to the Joint Committee on Power Diffraction Standards (JCPDS) data files (jarosite [no. 22-0827], jarosite [no. 26-1014], calcium sulfate [CaSO_4_·2H_2_O] crystallization [no. 33-0311], and goethite [no. 76-2301]), the sediments were found to be composed of jarosite, calcium sulfate, and goethite. However, the weak XRD peaks of schwertmannite particles may be masked by the peaks of higher-strength crystalline minerals such as jarosite and goethite. Jarosite-rich sediments occurred as pseudocubic or polygonized crystals at pH values of 1.8–3.0 in 1.8J, 1.8J10, 1.8J30, 2.3J, 2.3J10, 2.8J, and 2.8J10 systems. The crystallization of calcium sulfate was observed in 1.8J30, 1.8J50, 2.3J10, 2.3J30, 2.3J50, 2.8J30, and 2.8J50 systems, and goethite formed in the 2.8J10 system. 

**Figure 9:**
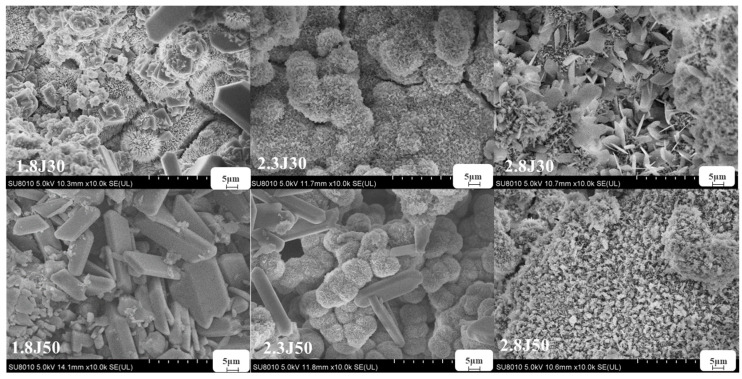
SEM images of the carbonate rock surfaces of the different systems (magnified 10,000×).

The sediments were characterized by a progressive transition from low crystalline assemblages composed of calcium sulfate and subordinated jarosite, to well crystal-line assemblages composed of jarosite, calcium sulfate, and goethite. When the hydrolysis of Fe^3+^ releases H^+^ to reduce pH (pH < 3) and monovalent cations are present in the solution, schwertmannite transforms into jarosite [43]. Based on the XRD analysis of 2.8J10 and the range of pH variation, the spherulitic aggregate (Figure 7) was goethite, which may have retained the schwertmannite or mineral crystal form of schwertmannite during the transformation of jarosite or schwertmannite into goethite [44]. As per the XRD analysis, calcium sulfate in the 2.3J30, 2.3J50, 2.8J30, and 2.8J50 systems was thick, plate-like, and needle-like, while that on the rock surfaces of 2.8J30 and 2.8J50 (Figure 9) was mainly flaky and needle-like. The growth of calcium sulfate crystals is inhibited by Ca^2+^, Mg^2+^, and Fe^3+^ in the solution [45], which affects the morphology and size of gypsum crystals [46]. 

The range of pH variation, oxidation rate of Fe^2+^, removal rate of TFe, and main secondary mineral categories of the different systems are shown in Table 2. In this study, significant chemical and mineralogical differences occurred due to variations in the initial pH and dosage of carbonate rock. Compared to other systems with different initial pH, in the systems of 1.8J30,2.3J10, and 2.8J10, the TFe removal significantly increased and the changes of pH were conducive to the formation of secondary minerals or to form the new secondary mineral phase. Liu et al. [47] also found that with the formation of secondary Fe minerals with a mixed schwertmannite and jarosite, total Fe precipitation efficiency increased. According to previous research, the pH value and pH gradient between the surface of the mineral and the surrounding aqueous solution are key factors affecting the formation and phase transformation of secondary minerals [48].

## 4. Conclusions

The following conclusions could be drawn from this study.

(1) In this study, variations in pH and Ca^2+^ concentrations were found to affect the formation and phase transformation of secondary iron minerals. In the systems with an initial pH value of 1.8, 2.3, and 2.8, the optimum dosage of carbonate rocks was 30, 10, and 10 g, respectively. The oxidation rates of Fe^2+^ all reached 99% and the final removal rate of TFe in the 1.8J30 system was 67.37%, being 28.03% higher than that in 1.8J (39.34%), and the final removal rates of TFe in 2.3J10 and 2.8J10 were 55.63% and 61.82%, respectively, being 16.99% and 13.1% higher than those in 2.3J (38.64%) and 2.8J (48.72%), respectively.
(2) The number of sediments generated by adding carbonate rocks were significantly higher than those generated without carbonate rock addition. The 1.8J30, 2.8J10, and 2.8J30 were the highest sediments produced in different initial pH systems, respectively. In particular, 1.8J30, which generated 36.9 g·L^−1^ of sediment, was higher than that of 1.8J (6.6 g·L^−1^). The sediments were characterized by a progressive transition from low crystalline assemblages composed of calcium sulfate and subordinated jarosite, to well crystal-line assemblages composed of jarosite, calcium sulfate, and goethite.
(3) As a consequence, based on the initial pH value, the number of carbonate rocks added can be reasonably selected to control the rates of pH rise. When the pH is raised to an appropriate value, the removal rate of TFe, the mineral production, and the types of secondary minerals can be significantly improved. The findings of this study are of interest for engineering applications that consider combined microbial and carbonate rock treatments for AMD in karst areas.

## Figures and Tables

**Table 1:** Different initial pH treatment systems.

Groups	Initial pH of Systems	Carbonate Rock Dosage/g	Note
1.8J	1.8	0	Treatment ① was used to regularly monitor the pH value in the reaction system. The leachate samples were collected at specified intervals from the bottom outlet of syringe columns, filtered (0.22 μm), and the concentrations of Ca^2+^, Fe^2+^, and TFe in the reaction system were measured. Treatments ② and ③ were treated and filtered by vacuum suction, and the sediments were obtained by culture filtration and then dried in an oven at 50 °C to analyze the amount of sediment and mineral composition.
1.8J10	1.8	10	
1.8J30	1.8	30	
1.8J50	1.8	50	
2.3J	2.3	0	
2.3J10	2.3	10	
2.3J30	2.3	30	
2.3J50	2.3	50	
2.8J	2.8	0	
2.8J10	2.8	10	
2.8J30	2.8	30	
2.8J50	2.8	50	

**Table 2:** Range of pH variation, Fe^2+^ oxidation, TFe removal, sediment production, and main secondary minerals in the different systems.

Number	Range of pH Variation	Fe^2+^ Oxidation (%)	TFe Removal (%)	Sediment Production(g/L)	Main Secondary Minerals
1.8J	1.8–2.19	99.11	39.34	6.6	Jarosite
1.8J10	1.8–2.65	99.17	41.58	19.8	Jarosite
1.8J30	1.8–2.89	99.30	67.37	36.9	Jarosite and calcium sulfate
1.8J50	1.8–3.84	80.91	50.12	19.2	Calcium sulfate
2.3J	1.99–2.53	99.17	38.64	17.6	Jarosite
2.3J10	1.96–3.12	99.23	55.63	26.6	Jarosite and calcium sulfate
2.3J30	2.3–3.81	81.43	45.00	25.6	Calcium sulfate
2.3J50	2.3–3.92	82.21	48.83	24.7	Calcium sulfate
2.8J	1.87–2.8	99.17	48.72	26.8	Jarosite
2.8J10	2.03–3.48	99.26	61.83	31.9	Jarosite, calcium sulfate, and goethite
2.8J30	2.8–3.86	83.19	45.85	23.7	Calcium sulfate
2.8J50	2.8–3.99	85.18	50.38	30.1	Calcium sulfate

## Data Availability

The authors declare that [the/all other] data supporting the findings of this study are available within the article.
